# Intravascular embolization of a direct orbital arteriovenous fistula: case report and review of the literature

**DOI:** 10.3389/fopht.2025.1666024

**Published:** 2025-11-07

**Authors:** Olivia T. Cheng, Stella Y. Chung, Jeffrey M. Wilseck, Alon Kahana

**Affiliations:** 1 Corewell Health William Beaumont University Hospital Eye Institute, Corewell Health, Royal Oak, MI, United States; 2 Department of Ophthalmology, New York University (NYU) Langone Health, New York, NY, United States; 3 Kahana Oculoplastic and Orbital Surgery, Livonia, MI, United States

**Keywords:** orbital arteriovenous fistula, intraorbital arteriovenous fistula, orbital decompression, direct orbital arteriovenous fistula, orbital vascular lesions

## Abstract

Orbital arteriovenous fistulas are exceedingly rare and present a unique challenge due to difficulties with access. We report a case of a patient with an acute progressive direct orbital arteriovenous fistula causing orbital compartment syndrome and compressive optic neuropathy. He underwent medial orbital decompression followed immediately by direct cannulation of the vascular anomaly, through which two separate fistulas were embolized under fluoroscopic guidance.

## Introduction

Orbital arteriovenous fistulas are rare, high-flow vascular malformations characterized by a direct connection between the ophthalmic artery and one of the draining ophthalmic veins. They may arise spontaneously or in association with trauma (1). They are distinguished from orbital arteriovenous malformations by the lack of a central nidus (2). Given the rarity of this entity and the limited number of case reports published on its management, no standard treatment approach has been established. Herein, we review the literature and present a case of a rapidly progressive orbital arteriovenous fistula causing compressive optic neuropathy that was successfully treated with medial orbital decompression followed immediately by direct-access embolization of multiple independent fistula tracks using intraoperative catheter repositioning. The collection and evaluation of protected patient health information were Health Insurance Portability and Accountability Act (HIPAA)-compliant and adhered to the tenets of the Declaration of Helsinki.

## Case presentation

**Figure 1:**
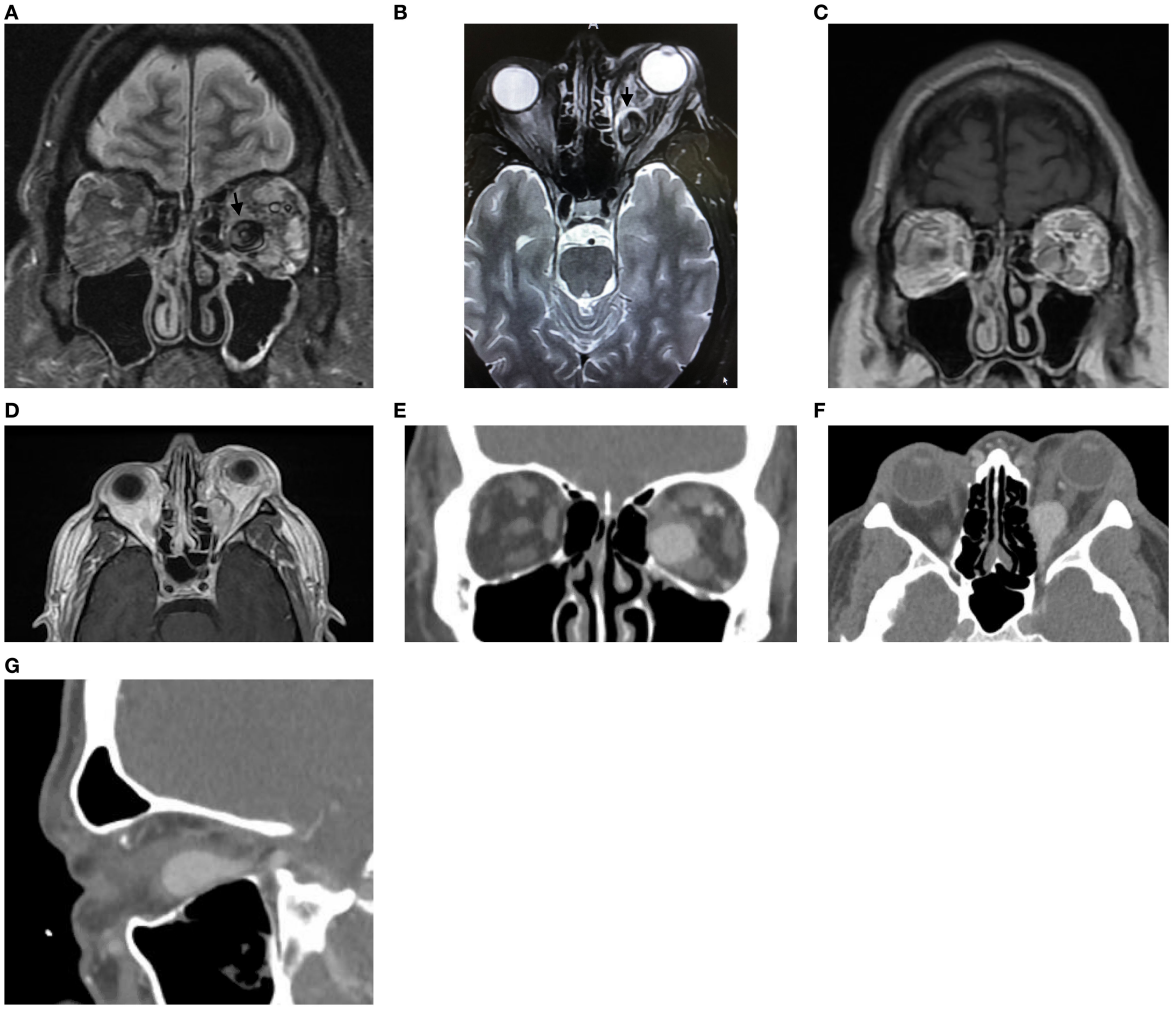
**(A)** Coronal transverse relaxation time short tau inversion recovery magnetic resonance image demonstrating a large venous aneurysm in the inferomedial left orbit. **(B)** Axial transverse relaxation time short tau inversion recovery magnetic resonance image demonstrating a dilated venous aneurysm in the posterior medial left orbit (arrow) with associated edema of the medial and lateral rectus muscles. **(C, D)** Longitudinal relaxation time post-gadolinium magnetic resonance coronal **(C)** and axial **(D)** images demonstrating enhancement of the venous aneurysm in the inferomedial left orbit. **(E**–**G)** Computed tomography angiography with intravenous contrast in coronal **(E)**, axial **(F)**, and sagittal **(G)** views demonstrating enhancement of the venous aneurysm, which extends to the orbital apex.

**Figure 2:**
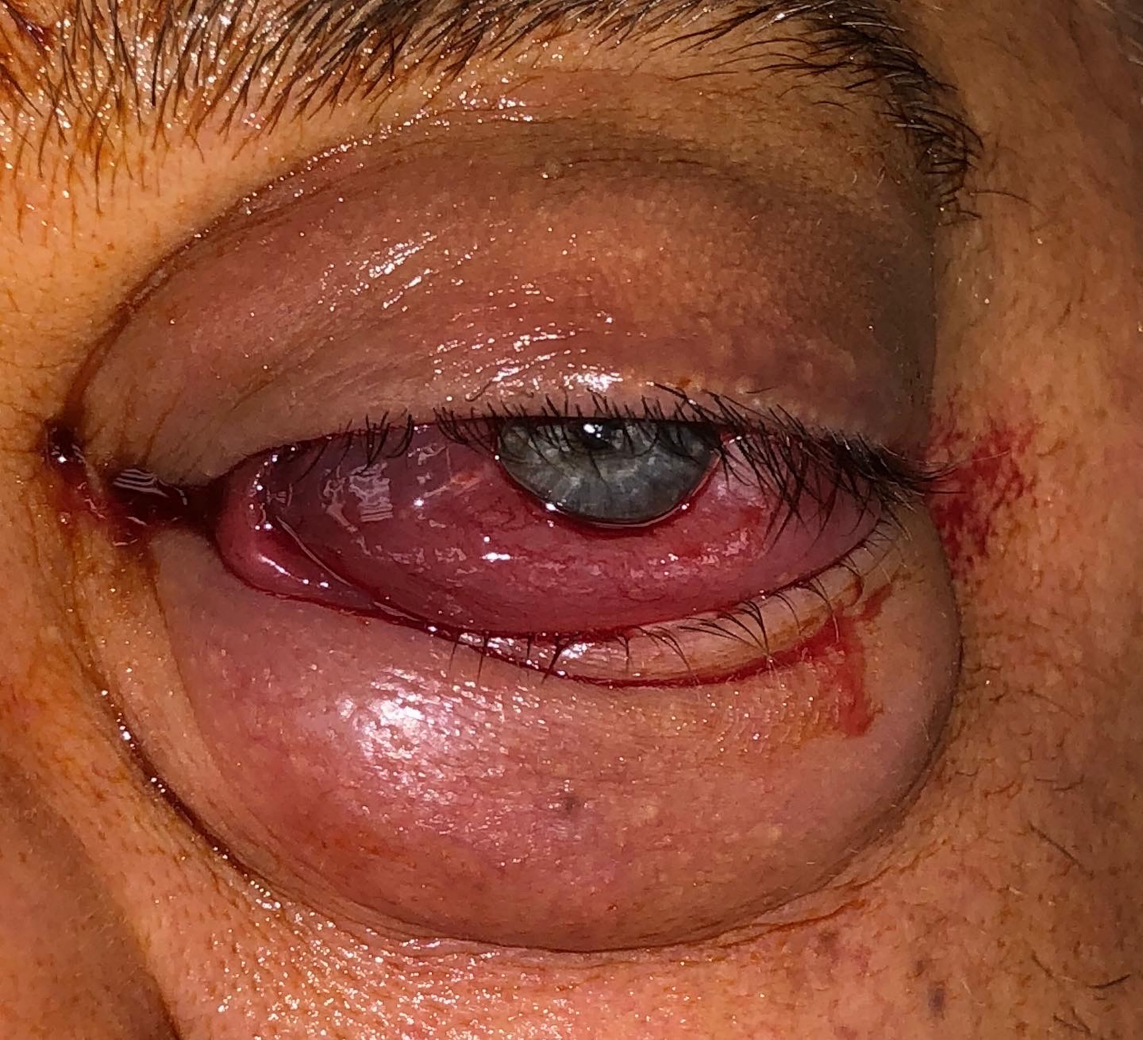
Pre-operative external photograph of the left eye demonstrating significant orbital congestion and chemosis. Photograph was taken on the day of intervention.

**Figure 3:**
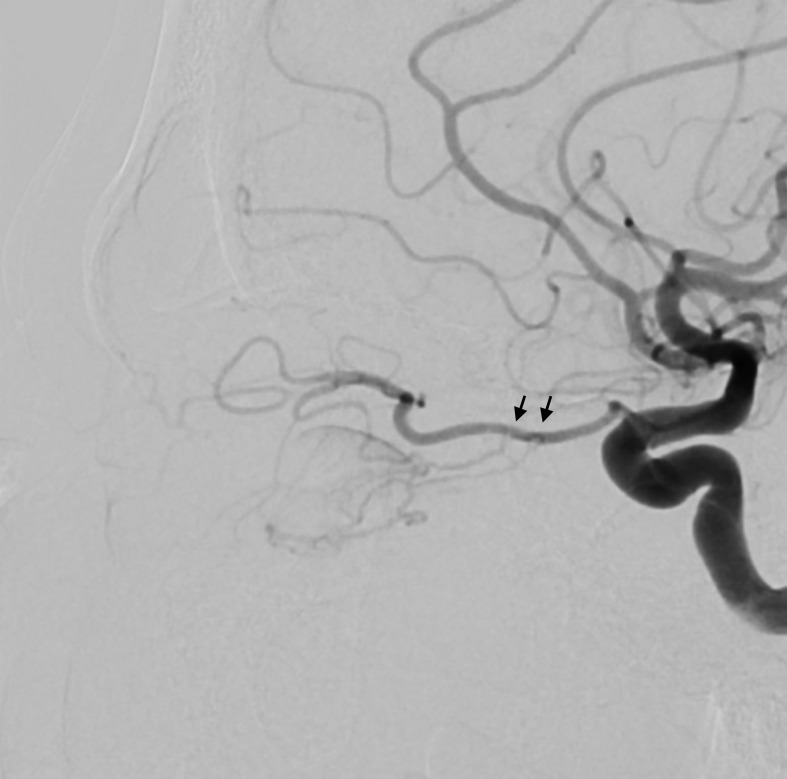
Lateral view of the angiogram after internal carotid artery injection demonstrating feeding arterioles off the proximal ophthalmic artery (arrows).

**Figure 4:**
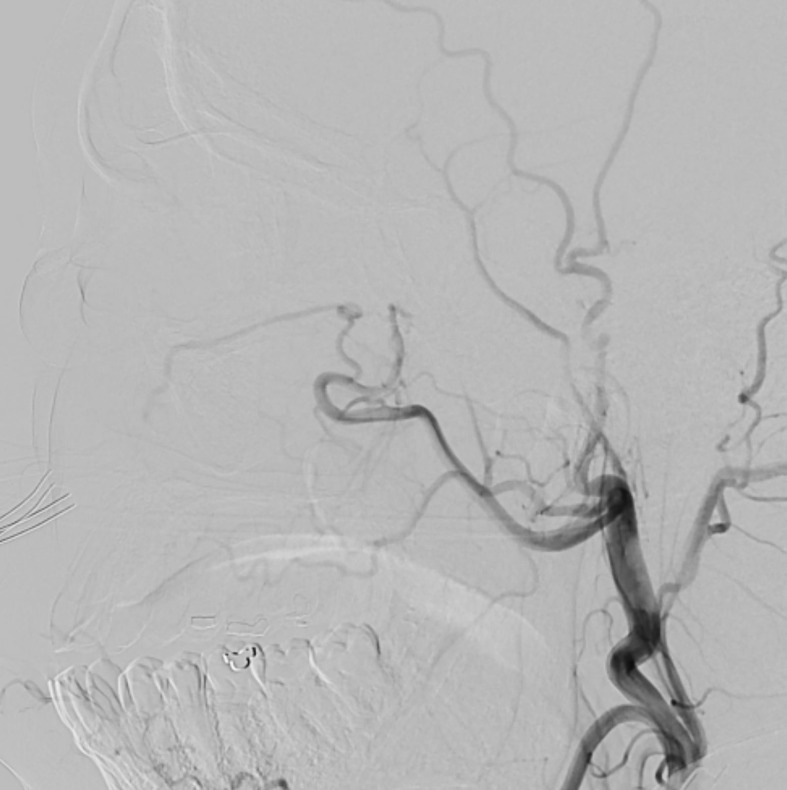
Lateral view of the angiogram after external carotid artery injection demonstrating no feeding of the venous aneurysm.

A 48-year-old man presented with 1 month of blurry vision in the left eye without a history of trauma. His past medical history was significant for thyroid cancer in remission and hypothyroidism. On presentation to outside ophthalmology, he was noted to have left optic neuropathy with decreased vision (20/100), poor color vision, relative afferent pupillary defect, restricted extraocular motility, chemosis, eyelid edema, and elevated intraocular pressure. He had 5 mm of left-sided relative proptosis on Hertel exophthalmometry. Imaging, both CT and MRI, was initially read as a varix (**Figure 1**). He was treated for possible orbital cellulitis and possible thyroid eye disease with intravenous antibiotics and intravenous methylprednisolone, respectively, with no improvement. His proptosis and visual function worsened rapidly (**Figure 2**), and he was referred to our practice. Reassessment of his imaging raised doubts regarding the diagnosis, and an urgent angiogram was recommended. He was admitted, and six-vessel angiography demonstrated a left orbital arteriovenous fistula fed by three direct branches of the left ophthalmic artery with drainage to the superior ophthalmic vein (SOV) and no communication with the cavernous sinus (**Figures 3**, **4**). MRI of the brain and orbits, and magnetic resonance angiography (MRA) and magnetic resonance venography (MRV) of the brain, demonstrated no evidence of vessel thrombosis or any previous connection to the cavernous sinus or other vasculature. Given the origin of the feeding arterioles off the proximal ophthalmic artery, an arterial approach was deemed to be high risk. Since the venous component of the fistula did not communicate with the cavernous sinus, this approach was not possible. A transvenous approach through the facial vein was also considered, but the indirect access would have made it difficult to guarantee occlusion at the fistula site or sites.

**Figure 5:**
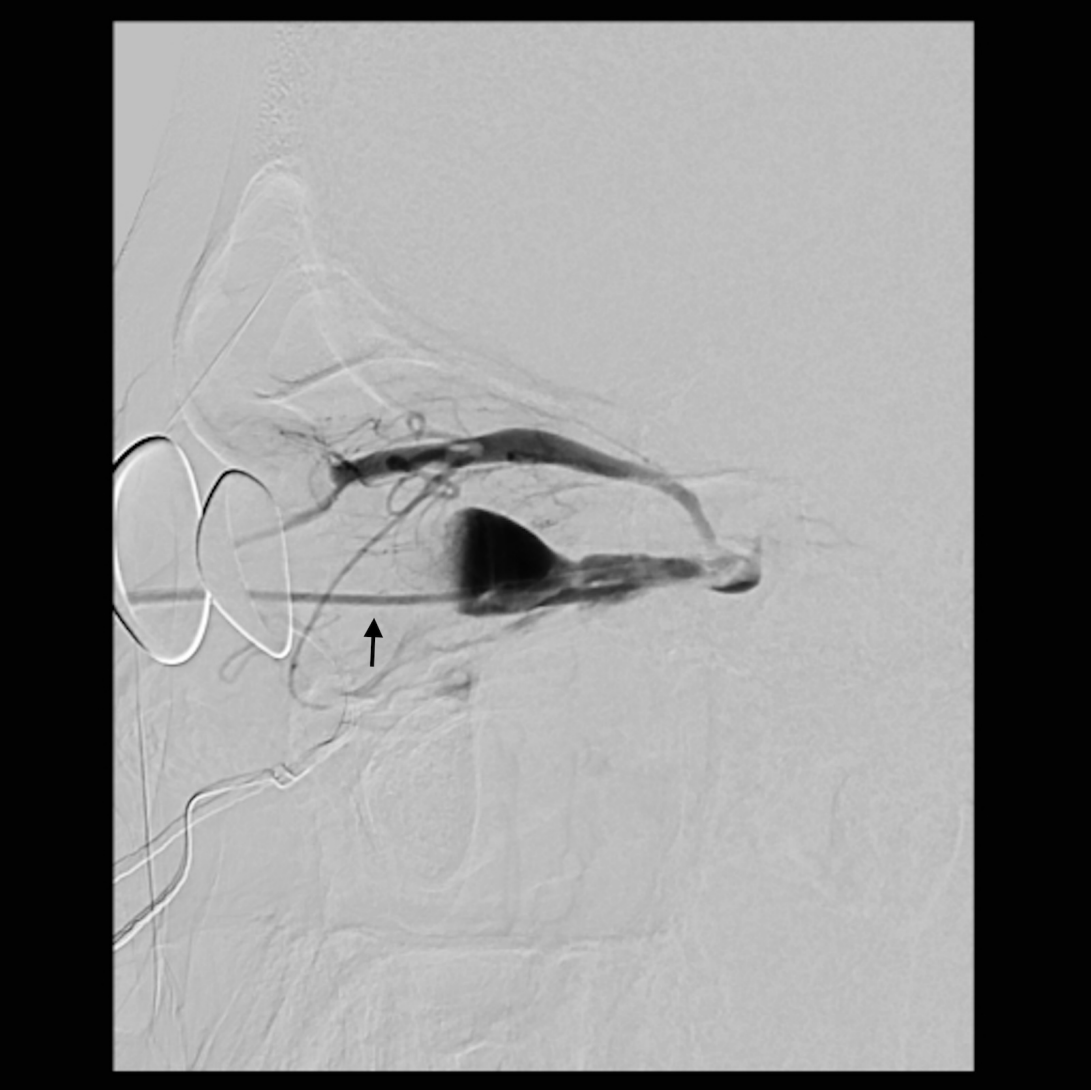
Lateral view of the angiogram demonstrating a percutaneously placed catheter (arrow) in the dilated venous aneurysm of the fistula. There is contrast outflow superficially without direct connection to the cavernous sinus.

**Figure 6:**
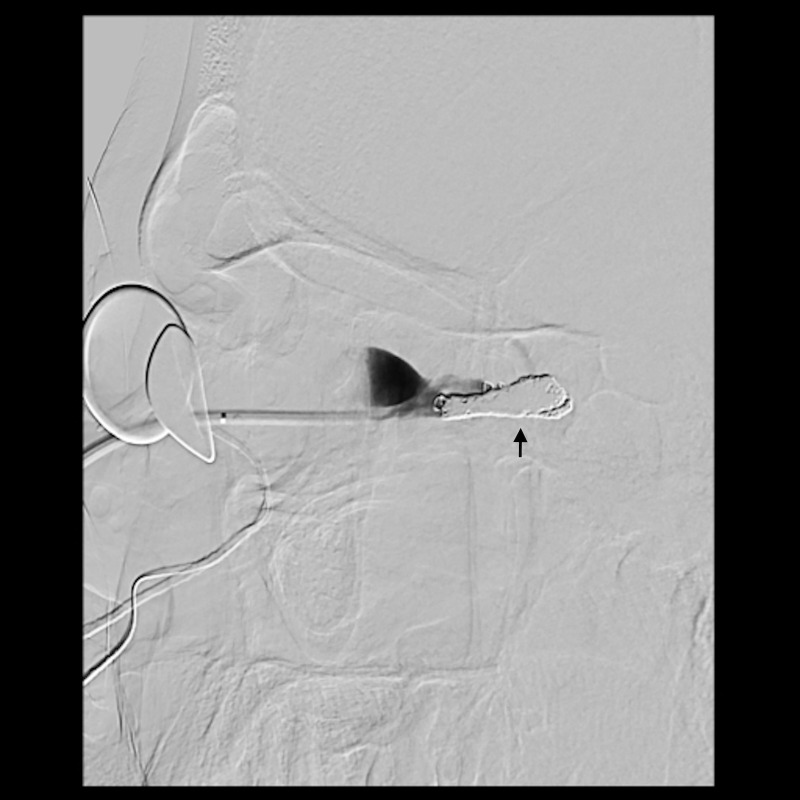
Lateral view of the angiogram after coiling of the venous outflow tracts of the fistula demonstrating complete occlusion of the outflow tracts. Arrow indicates location of coils.

A multi-disciplinary surgical plan was developed. The patient underwent left medial orbital decompression to decompress the optic nerve, decongest the orbit, and provide access to the venous bulb of the fistula. This was followed immediately by cannulation of the venous anomaly in the operating room using a 5-French micropuncture sheath under direct intraoperative observation (**Figure 5**), followed by transfer to the fluoroscopy suite. With intraoperative angiography providing a “road map”, the venous outflow channel to the SOV was embolized with 20 coils, revealing a second outflow channel. The orbital catheter was manipulated and repositioned to access the second channel, which was then embolized with another 18 coils. At this point, the venous component was noted to have venous stasis (**Figure 6**), which would promote clotting followed by involution of the venous anomaly. The SOV was preserved and patent. Given the potential for a compartment syndrome, a lateral canthotomy and a cantholysis were performed preemptively to preserve normal intraocular and intraorbital pressure in the immediate post-operative period.

The patient was admitted to the intensive care unit (ICU) for 24 hours and recovered well in the first few days following surgery. At 1 week post-operatively, he was found to have severe swelling and congestion of the orbit with more than 2 cm of proptosis and mild optic disc edema, but intraocular pressure was only mildly elevated to 24 mmHg. He was started on an oral prednisone taper. At 3 weeks post-operatively, he was recovering well with resolved disc edema, improved proptosis, visual acuity of 20/30, improved extraocular motility, and a trace afferent pupillary defect. At long-term follow-up, his visual acuity was restored with normal color vision, no afferent pupillary defect, and no proptosis.

## Discussion

Orbital arteriovenous fistulas can cause vision-threatening compartment syndrome, leading to compressive optic neuropathy and ophthalmoplegia. Clinical presentation is characterized by signs and symptoms of orbital venous congestion and may mimic that of an orbital varix, carotid-cavernous fistula, orbital tumor, orbital cellulitis, or thyroid eye disease. The most commonly reported presenting symptoms include proptosis, chemosis, double vision, and vision loss (3). Definitive diagnosis is made by cerebral angiography, which is also essential for treatment planning (1, 4). The decision to obtain an angiogram requires a high level of suspicion and familiarity with the differential diagnosis. A team of neuro-radiologists and orbital specialists is key to achieving diagnostic accuracy.

Most recently, Pathuri et al. published a review of the literature on this subject, organized by treatment modality, and Krylova and Hauck published a review of the literature on this subject, including dural arteriovenous fistulas (2, 5). The authors performed a literature search of PubMed from 1978 to August 2025 for cases of pure intraorbital arteriovenous fistulas with documented intervention and outcome. Dural arteriovenous fistulas and arteriovenous malformations were excluded. Among 29 identified cases, including the present case, 14/29 (48%) were treated with transvenous embolization alone (2, 3, 6–8), 7/29 (24%) were treated with orbitotomy combined with direct fistula treatment (9–14), 4/29 (14%) were treated with craniotomy (15–18), and 4/29 (14%) were treated with transarterial embolization (18–21). Cases in which orbitotomy was performed are summarized in **Table 1**. In addition, we noted 18 reported cases of spontaneous resolution spanning a variety of etiologies (2, 18), although 5/18 (28%) cases had reported visual decline. Given the limited published experience with this rare entity, clear indications for observational management are yet to be defined.

**Table 1:** Intraorbital arteriovenous fistulas treated with orbitotomy from 1978 to August 2025.

Study	Age/Sex	Presentation	Pathogenesis	Treatments	Results	Complications	Outcome
Wigton et al., 2012 (14)	61/M	6 months of decreased vision and periorbital edema	Spontaneous	Lateral orbitotomy with direct cannulation of SOV; SOV noted to have thrombosed following intraoperative angiography; distal SOV ligated with aneurysm clip	Full resolution	None	Stable at 4 months
Naqvi et al., 2013 (9)	72/M	6 months of progressive proptosis, chemosis, and injection	Spontaneous	Orbitotomy via upper eyelid crease with direct cannulation of SOV and embolization	Full resolution	None	Stable at 6 months
Wang et al., 2016 (10)	81/F	3 weeks of proptosis, eyelid edema, and chemosis	Spontaneous	1. Transvenous embolization via petrosal sinus attempted but could not be advanced 2. Orbitotomy with direct cannulation of SOV and embolization	Full resolution	None	Stable at 1 month
Robledo et al., 2023 (11)	Not given	10 days of vision loss, proptosis, and chemosis	Not given	1. Transvenous embolization via anterior facial and angular veins unsuccessful 2. Orbitotomy via subciliary incision with stereotactic-guided direct puncture and embolization, followed by endoscopic medial orbital decompression	Full resolution	None	Improved symptoms immediately after procedure; no long-term follow-up reported
Ghahvehchian et al., 2023 (12)	25/M	3 years of proptosis, ocular pain, and injection	Spontaneous in the setting of ipsilateral congenital fronto-orbital lymphaticovenous malformation	1. Two attempted endovascular treatments unsuccessful 2. Endoscopic-assisted orbitotomy with bipolar electrocauterization of the AVF followed by orbital decompression	Full resolution	None	Stable at 6 months
Choi et al., 2024 (13)	75/M	Proptosis, injection, tenderness, and diplopia (duration not given)	Spontaneous	1. Transarterial embolization of adjacent vessels as temporizing measure 2. Transconjunctival orbitotomy with direct cannulation of vascular anomaly and embolization	Full resolution	None	Improved symptoms at 2 weeks
Present case	48/M	1 month of progressive optic neuropathy, proptosis, and chemosis	Spontaneous	Medial orbital decompression with direct cannulation of vascular anomaly and embolization of two fistulas	Full resolution	None	Stable at 2 months

Cases are listed chronologically with details of presentation, treatment, and outcome.

SOV, superior ophthalmic vein; AVF, arteriovenous fistula.

With the advancement of endovascular techniques over the past two decades, transvenous embolization has become the preferred method of management, when possible (22). When transvenous embolization fails or is not anatomically possible, direct surgical exposure can allow an alternative venous access for embolization (2, 9–13, 17). Notably, orbital decompression combined with direct surgical access for the transvenous management of orbital arteriovenous fistula has been described in three previous cases with successful treatment outcomes (2, 11, 12).

In the present case, given the high-risk location of the three arterial feeding branches, the degree of orbital congestion and optic neuropathy, and the rapidity of progression, the surgical plan was designed for the best chances of safe, effective, and definitive management of the fistula combined with rapid relief of the compressive optic neuropathy. The surgical decompression of the orbital apex prior to embolization provided several benefits: 1) it reduced pressure on the optic nerve, 2) it improved venous outflow to reduce congestion, 3) it provided additional space for the surgical exploration of the arteriovenous fistula, and (4) it accounted for the anticipated increase in orbital inflammation and congestion following a surgical procedure and vessel embolization. Following the procedure, prophylactic lateral canthotomy and superior and inferior cantholysis were performed to further reduce the effects of post-operative congestion. One week after the procedure, the patient had particularly pronounced orbital edema that may have been similar to the phenomenon of “paradoxical worsening” observed to follow thrombosis or embolization of arteriovenous malformations contiguous with the cavernous sinus (23). The multidisciplinary approach adopted in this case resulted in rapid resolution of the compressive optic neuropathy and associated proptosis as well as the maintenance of safe intraocular pressures throughout the post-operative course, without complications. In certain cases, combining surgical decompression with endovascular embolization may provide the best chance of a good outcome in treating the rare direct orbital arteriovenous fistula.
